# The first validation of the Functional Assessment of Cancer Therapy Hepatobiliary (FACT-Hep) for evaluating health-related quality of life (HRQOL) in patients with advanced-stage intrahepatic cholangiocarcinoma (biliary tract cancer)

**DOI:** 10.1371/journal.pone.0321618

**Published:** 2025-04-28

**Authors:** Teerachat Saeheng, Nisit Tongsiri, Kesara Na-Bangchang

**Affiliations:** 1 Center of Excellence in Pharmacology and Molecular Biology of Malaria and Cholangiocarcinoma, Chulabhorn International College, Thammasat University (Rangsit Campus), Klongneung District, Pathumthani Province, Thailand; 2 Sakol Nakorn Hospital, Muang District, Sakol Nakorn Province, Thailand; 3 Drug Discovery and Development Center, Office of Advanced Science and Technology, Thammasat University (Rangsit Campus), Klongneung District, Pathumthani Province, Thailand; National Research Centre, EGYPT

## Abstract

**Background:**

The FACT-Hep questionnaire has been used to evaluate the health-related quality of life (HRQOL) in various types of hepatobiliary cancer. Nevertheless, the application in intrahepatic cholangiocarcinoma (iCCA) has been limited. The study aimed to validate the applicability of FACT-Hep as a reliable tool for evaluating HRQOL in patients with advanced-stage iCCA.

**Methods:**

*Atractylodes lancea* capsules were tested for efficacy and safety in a randomized, controlled phase IIA. Internal consistency, intraclass correlation, test-retest reliability, discriminant and convergent validity, and FACT-Hep score-clinical response connection were assessed.

**Results:**

Thirty-nine patients qualified for the research. Cronbach’s alpha coefficients > 0.7 showed internal consistency for all subscale items from baseline (day 1) to 90 days. For ICC, convergent, and discriminant validity, all items except the HepCS subscale were reliable. Patients with ECOG scores of 0–1 or 2 had significantly different HepCS subscale values—calculated effect size for subscale score change over time. FACT-G and FWB differed significantly in low-dose group 1. At 3-month, 1-month, and 2-month follow-ups, Group 2 (high dose) patients had significant differences in FACT-Hep, TOI, and HepCS. The effect size was the same in Group 3 (untreated). Group 1 and 2 non-progressive patients exhibited lower FWB and EWB scores than progressive patients. The treated survivors had lower FACT-Hep, TOI, HepCS, FWB, and EWB scores than the non-treated survivors, which was linked to AL treatment side effects.

**Conclusion:**

The FACT-Hep detects changes and is a reliable and valid tool for assessing HRQOL in patients with advanced-stage iCCA.

## Introduction

Assessing the quality of life (QoL) in patients undergoing chemotherapy is essential for understanding the disease’s symptoms and the treatment’s adverse effects. QoL assessments cover various domains, including physical, emotional, and social well-being [1].

The Functional Assessment of Cancer Therapy-Hepatobiliary (FACT-Hep) is a health-related quality of life (HRQOL) tool specifically designed to evaluate the functional outcomes of patients undergoing treatment for hepatobiliary cancers [2–27]. It includes five key components: emotional well-being (EWB), functional well-being (FWB), social/family well-being (SWB), physical well-being (PWB), and a hepatobiliary cancer subscale (HepCS). The HepCS subscale contains questions that precisely assess symptoms related to hepatobiliary carcinoma and treatment side effects [2–27].

While FACT-Hep was originally developed for hepatobiliary cancers, its primary validation and use have focused on hepatocellular carcinoma [2–27]. Its clinical application in cholangiocarcinoma (CCA) [17,25], especially in intrahepatic cholangiocarcinoma (iCCA), has been more limited [17,25]. In CCA, FACT-Hep has been utilized to assess the HRQOL of patients undergoing chemotherapy and endoscopic retrograde cholangiopancreatography (ERCP) [17,25,28–31]. However, the tool’s validity for evaluating HRQOL in CCA patients treated with herbal medicine or other therapies for biliary tract cancers remains unexamined.

Alongside FACT-Hep, the EORTC QLQ-BIL21 and EORTC QLQ-C30 questionnaires have been used to assess HRQOL in CCA patients [1,32]. These tools have been validated for use in CCA and gallbladder cancer, making them reliable for measuring HRQOL after various treatments.

Given the importance of having a robust tool to guide treatment decisions, this study aimed to assess the validity and reliability of FACT-Hep in patients with advanced-stage iCCA.

## Methods

### Study design, patients, and treatment

The study was part of a single open-labeled, randomized, controlled phase 2A clinical trial conducted in 2020 and 2021 at Sakon Nakorn Hospital, Sakon Nakorn, Thailand.^33^ The recruitment period was from 1 February 2021–30 April 2021. The study protocol was approved by Sakon Nakorn Hospital (No. 049/2563) (049/2020) before the recruitment period before conducting the study. It was noted that the ethical approval covered the study period. The ethical approval for this study was approved by the Sakon Nakorn ethical committee. The 2020 (English version) and 2563 (Thai version) are the same year, where 2020 is A.D. (Anno Domini), and 2563 is BE (Buddhist Era) (2563–543 = 2020 for A.D.). The study was performed in compliance with Good Clinical Practice (GCP) guidelines and the Declaration of Helsinki [33]. Written informed consent was obtained from each participant before enrolment. The registration number was TCTR20210129007 (Thai Clinical Trials Registry) [33]. Patients were randomized into three groups using Box randomization for allocation. Thirty or at least 12 patients in each group were adequate for a statistical analysis in phase 2A clinical trial [33].

The patient’s inclusion and exclusion criteria were described in the supplementary materials.

### Evaluation of treatment outcomes

#### Questionnaires.

The HRQOL in patients with advanced-stage iCCA were assessed using FACT-Hep and its subscales, *i.e.,* FACT-G. FACT-G is generally used in cancer therapy and consists of 27 items, *i.e.,* PWB (7 items), SWB (7 items), EWB (6 items), and FWB (7 items). FACT-Hep (18 items) includes an additional specific questionnaire for hepatobiliary cancer (HepCS), hepatocellular carcinoma (HCC) and biliary tract cancers. The three components, PWB, FWB, and HepCS, which are related to physical/functional activities for a clinical outcome is the Trial Outcome Index (TOI). FACT-Hep (FACT-G plus HepCS) questionnaires and guidelines have been translated into Thai (FACT-Hep version 4.0). The HRQOL scores were evaluated at baseline (day 1) and on days 14, 28 (1 month), 56 (2 months), 90 (3 months), and 120 (4 months) after treatment. Data are reported as mean ± 95% confidence interval (CI) or median (interquartile or IQR) values. Patients were required to complete the assessment at all time points.

#### Clinical responses.

Progression-free survival rate (PFSR), disease control rate (DCS), tumor growth, and overall survival (OS) were evaluated in all groups at 4 months (120 days) according to RECIST Version 4.0 guideline for solid tumor treatment. In addition, ECOG was used to assess the patient’s performance [32]. Associations between clinical response and the scores of FACT-Hep, FACT-G, TOI, and HepCS were evaluated.

### Methods of data analysis

The details of the methods of data analysis and the effect of size are described in supplementary materials.

## Results

### Patient enrolment

**Fig 1:**
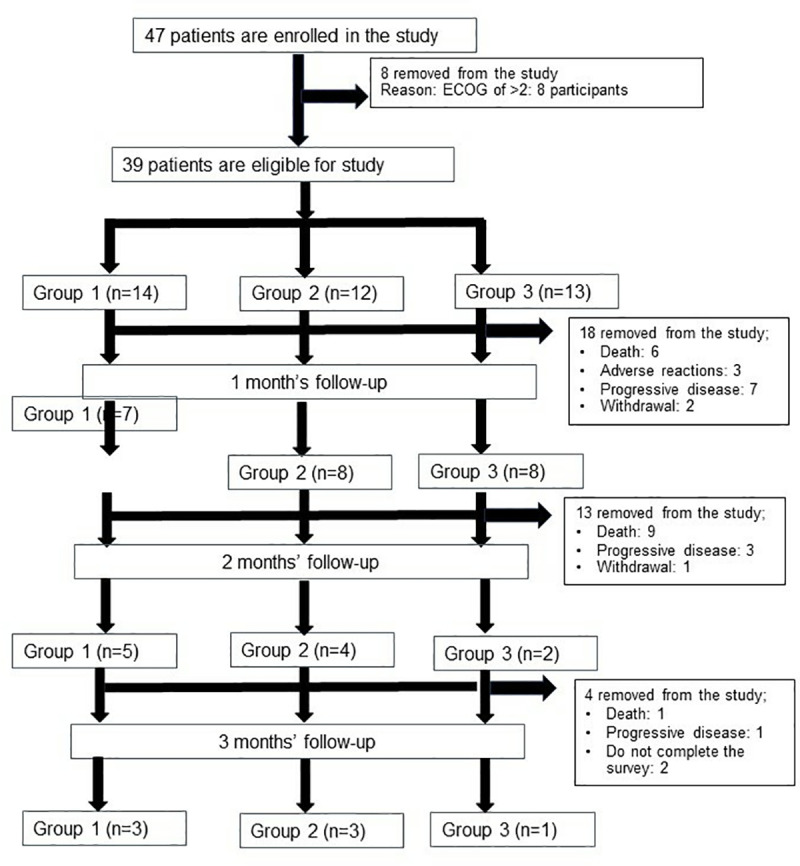
A flow chart diagram of the study.

Forty-seven patients with advanced-stage iCCA were recruited in the three-arm randomized control trial. Thirty-nine patients were eligible for the analysis of baseline FACT-Hep and ECOG performance and the consistency and reliability of the FACT-Hep questionnaire (n=14, 12, and 14 patients for Groups 1, 2 and 3, respectively). A flow chart diagram of the study is shown in **Fig 1**.

### Questionnaire completion

All 39 patients completed questionnaires at the baseline assessment. Eighteen patients did not complete the study on day 28 (6 deaths, 3 with adverse events, 7 with disease progression, and 2 withdrawn cases). Thirteen patients did not complete the study on day 56 (9 deaths, 3 with disease progression, and 1 withdrawn case). Four patients did not complete the study on day 90 (1 death, 1 with disease progression, and 2 withdrawn cases). One patient did not complete the study on day 120 (1 death). At the end of the study (120 days), only 6 patients were available to assess FACT-Hep on day 120.

### FACT-Hep scale structure: consistency and reliability

The internal consistency of the FACT-Hep questionnaires was calculated for each scale at baseline and on days 28, 56, 90, and 120 of treatment. The respective overall Cronbach’s alpha coefficients [median (range)] were 0.814 (0.80–0.813), 0.772 (0.748–0.782), 0.753 (0.721–0.769), 0.282 (0.123–0.412), and 0.558 (0.486–0.608) (Table 1). At baseline and on days 28 and 56, all Cronbach’s alpha coefficients in subscales, i.e., FACT-G, TOI, HepCS, FWB, SWB, EWB, and PWB, met the criteria of >0.7 (0.74–0.813). The Cronbach’s alpha coefficients for each item on each day are shown in S1 Table.

**Table 1:** The Cronbach’s alpha coefficients of FACT-Hep and its subscales at baseline, and on days 28 (1 month), 56 (2 months), 90 (3 months), and 120 (4 months) of treatment follow-up. Data are presented as mean ±95% CI.

Category	Baseline	1 month (28 days)	2 months (56 days)	3 months (90 days)	4 months (120 days)
**Physical well-being (PWB)**	0.808 (0.804–0.811)	0.761 (0.756–0.765)	0.747 (0.731–0.762)	0.218 (0.177–0.259)	0.524 (0.502–0.546)
**Social well-being (SWB)**	0.808 (0.805–0.812)	0.771 (0.767–0.775)	0.750 (0.747–0.751)	0.318 (0.264–0.371)	0.579 (0.565–0.594)
**Emotional well-being (EWB)**	0.813 (0.809–0.817)	0.770 (0.766–0.773)	0.740 (0.737–0.747)	0.271 (0.216–0.314)	0.536 (0.511–0.561)
**Functional well-being (FWB)**	0.812 (0.806–0.815)	0.769 (0.763–0.774)	0.753 (0.744–0.761)	0.324 (0.272–0.375)	0.585 (0.568–0.602)
**HepCS (Additional concerns)**	0.809 (0.806–0.813)	0.767 (0.761–0.773)	0.748 (0.741–0.755)	0.252 (0.220–0.283)	0.538 (0.517–0.557)

For the reliability of Cronbach’s alpha, the ICC values were assessed up to 90 days. Intraclass correlation (ICC) was evaluated for a test and re-test reliability at baseline and on days 28, 56 and 90. A two-way mixed effect model, single measurements, and absolute agreement were applied to calculate the ICC values. The ICC values at baseline and on days 28, 56 and 90 of treatment ranged from 0.427 to 0.795 (p <0.001). The items with ICC values > 0.5 were PWB (0.629), SWB (0.695), EWB (0.795), and FWB (0.701). Other ICC values for FACT-Hwp, FACT-G, TOI, HepCS and PWB were 0.441, 0.451, 0.454, and 0.427, respectively (**Table 2).**

**Table 2:** The ICC values for FACT-Hep and its subscales at baseline and 1, and 2 months’ follow-up (Data are presented as mean ± 95%CI).

Categories	Baseline and follow-up	P-value
**FACT-Hep**	0.441 (0.127–0.777)	0.002
**FACT-G**	0.451 (0.133–0.740)	0.002
**TOI**	0.454 (0.136–0.741)	0.002
**HepCS**	0.427 (0.108–0.725)	0.004
**PWB**	0.629 (0.344–0.840)	<0.001
**SWB**	0.695 (0.434–0.873)	<0.001
**EWB**	0.795 (0.590–0.92)	<0.001
**FWB**	0.701 (0.443–0.875)	<0.001

### Construct validity

**Fig 2:**
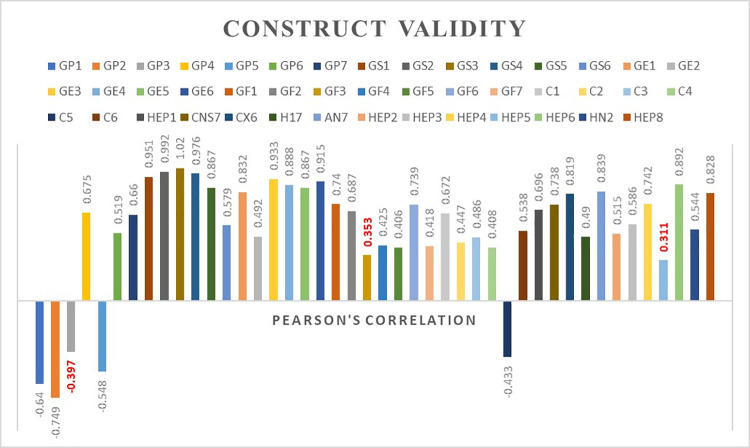
Construct validity (Pearson’s correlation of each item).

A total of 45 items were analyzed using Pearson’s correlation test. Based on factor analysis, three items (GP3, GF3, and HEP5) exhibited Pearson’s r correlations of less than 0.4 (Fig 2). For discriminant validity, the total number of comparisons for FACT-Hep was 2,025, with 876 violation comparisons identified. **Fig 2** displays the Pearson correlation coefficient (R²) for the FACT-Hep questionnaire, which includes 45 items across five domains: physical well-being (GP1–7), family/social well-being (GS1–7, Q1), emotional well-being (GE1–6), functional well-being (GF1–7), and additional concerns (C1-C6, Hep1–8, CNS7, Cx6, An7, H17, HN2). The R² value is used to assess construct validity by evaluating the strength and direction of the linear relationship between this newly developed measure and an established, related measure

### Sensitivity to change and effect size of FACT-Hep over time

Paired t-tests or Wilcoxon Ranked tests were used to analyze all FACT-Hep subscales at baseline and during follow-up. Significant changes in these subscales are detailed in Table 3.

**Table 3:** Comparisons of FACT-Hep scores and its subscales between baseline and follow-up (1, 2, 3 months’-follow-up).

Categories	Mean difference	Standard deviation	Standard errors	Mean difference (95% CI)			df	p-value	Effect size (ES)
**Lower bound**	**Upper bound**
**Group 1 (3 month follow-up)**									
**FACT-Hep**	−15.33	7.37	4.25	−33.64	2.97	−3.60	2	0.069	−5.02
**FACT-G**	−8.33	3.21	1.85	−16.32	−0.35	−4.49	2	0.046*	−2.37
**TOI**	−13.33	6.35	3.67	−29.11	2.44	−3.63	2	0.068	−3.52
**HepCS**	−7	6.24	3.60	−22.51	8.51	−1.94	2	0.192	−2.78
**FWB**	−5.67	1.15	0.67	−8.5351	−2.80	−8.5	2	0.014*	−0.12
EWB**	**–**	**–**	**1.837**	**–**	**–**	**-**1.08	1	0.276	-0.36
**SWB**	−3.67	3.51	2.03	−12.39067	5.05	−1.80	2	0.212	0.32
**PWB**	−0.67	2.08	1.20	−5.83781	4.50	−0.55	2	0.635	−1.49
**Group 2 (1-month follow-up)**									
**FACT-Hep**	−15	14.75	5.21	−27.33	−2.66	−2.87	7	0.024*	−0.96
**FACT-G**	−8.25	14.03	4.96	−19.98	3.48	−1.66	7	0.14	−0.563
**TOI**	−11.125	11.55	4.08	−20.78	−1.46	−2.72	7	0.03*	−1.17
**HepCS**	−6.75	7.51	2.65	−13.03	−0.46	−2.54	7	0.039*	−1.45
**FWB**	−2	6.34	2.24	−7.30	3.30	−0.89	7	0.40	−1.06
EWB**	–	5.895	–	–	–	0.17	15	0.86	−0.40
SWB**	–	4.71	–	–	–	0.53	13	0.59	−0.15
**PWB**	−2.375	4.24	1.49	−5.92	1.17	−1.58	7	0.15	−0.32
**Group 2 (2-month follow-up)**									
**FACT-Hep**	−19	9.96	4.98	−34.85	−3.140	−3.813	3	0.032*	−1.97
**FACT-G**	−5.5	10.24	5.12	−21.80	10.80	−1.07	3	0.362	−0.59
**TOI**	−21.25	11.23	5.62	−39.13	−3.37	−3.78	3	0.032*	−3.46
**HepCS**	−13.5	7.50	3.75	−25.44	−1.55	−3.59	3	0.037*	−6.48
**FWB**	−7	5.65	2.82	−16.00	2.00	−2.47	3	0.09	−0.22
EWB**	–	–	1.87	–	–	0	3	1	0.21
**SWB**	0.75	3.77	1.88	−5.25	6.75	0.39	3	0.718	0.21
PWB**	–	–	1.12	–	–	1.34	3	0.18	−1.53
**Group 2 (3-month follow-up)**									
**FACT-Hep**	−18	3.60	2.08	−26.96	−9.04	−8.64	2	0.013*	−2.05
**FACT-G**	−6.33	7.63	4.41	−25.31	12.63	−1.43	2	0.287	−1.56
TOI**	–	–	1.871	–	–	1.604	6	0.109	−9.24
**HepCS**	−13.67	8.32	4.8	−34.35	7.02	−2.84	2	0.105	−4.58
FWB**	–	–	1.871	–	–	1.604	6	0.109	−0.53
**EWB**	1	6.08	3.51	−14.11	16.11	0.28	2	0.803	–
**SWB**	2.33	3.51	2.03	−6.39	11.05	1.15	2	0.369	−0.35
**PWB**	−2.67	2.08	1.20	−7.84	2.50	−2.22	2	0.157	−1.73

*Statistical significant difference; **Wilcoxon Signed Rank Test

Group 1 patients observed significant differences on day 90 of follow-up in the FACT-G (p = 0.014, ES = -2.37) and FWB (p = 0.046, ES = -1.49) subscales.

For Group 2 patients, significant differences were detected as follows:

- On day 28 of follow-up: FACT-Hep (p = 0.024, ES = -0.97), TOI (p = 0.03, ES = -1.17), and HepCS (p = 0.039, ES = -1.45).
- On day 56 of follow-up: FACT-Hep (p = 0.032, ES = -1.97), TOI (p = 0.032, ES = -3.44), and HepCS (p = 0.037, ES = -6.48).
- On day 90 of follow-up: FACT-Hep (p = 0.013, ES = -2.05).

For Group 3 patients, no significant differences in FACT-Hep subscales were identified on days 28, 56, or 90 of follow-up.

### Clinically meaningful changes in FACT-Hep

The distribution-based methods were initially employed to guide the estimation of the MCIDs. SEM was used as it is theoretically less sample-dependent and has greater generalizability [34]. **Table 4** presents the Minimal Clinically Important Differences (MCID) values, which represent the smallest detectable clinical changes in treatment impacts on quality of life. These values help determine whether a therapy produces a significant improvement in a patient’s outcome from their perspective. The baseline MCIDs in Group 1 for FACT-Hep and its subscales measures ranged from 1.94 to 5.49. The FACT-Hep scores showed the highest MCID value of 5.49 and the FWB showed the lowest MCID value of 1.94. The MCID value for HepCS was 2.99. At the end of the study (day 120), the MCIDs of FACT-Hep and HepCS were significantly increased to 10.71 and 8.35, respectively. The baseline MCIDs in Group 2 patients ranged from 0.93 to 6.87. FACT-Hep showed the highest MCID value (6.87), while HepCS showed the lowest value (2.69). At the end of the study (day 120), the MCIDs for FACT-Hep and HepCS were 5.06 and 5.53, respectively. The corresponding values for Group 3 patients at baseline were 6.53 and 5.68, respectively. The patients in Group 3 (untreated group) were available only for 28 and 56 days of follow-up. Baseline MCIDs for FACT-Hep and HepCS in Group 3 patients were 6.53 and 3.68, respectively. The corresponding values on day 56 of follow-up were 11.25 and 4.09, respectively.

**Table 4:** Minimally important clinical difference (MICD) estimates.

Category	Criterion		
**1/3 SD**	**1/2SD**	**SEM**
**Group 1**			
**FACT-Hep**			
Baseline	4.23	6.3	5.49
1 month (28 days)	4.15	6.37	6.01
2 months (56 days)	4.01	6.01	5.80
**FACT-G**			
Baseline	3.08	4.06	4.03
1 month (28 days)	4.03	6.05	5.83
2 months (56 days)	1.26	1.89	1.82
**TOI**			
Baseline	3.68	5.52	4.82
1 month (28 days)	2.66	3.99	3.87
2 months (56 days)	4.1	6.15	5.95
**HepCS**			
Baseline	2.28	3.42	2.99
1 month (28 days)	1.57	2.35	2.27
2 months (56 days)	2.12	3.19	3.08
**PWB**			
Baseline	1.89	2.83	2.48
1 month (28 days)	1.28	1.92	1.88
2 months (56 days)	1.32	1.98	1.94
**SWB**			
Baseline	1.79	2.68	2.35
1 month (28 days)	2.12	3.18	3.05
2 months (56 days)	1.29	1.95	1.86
**EWB**			
Baseline	2.01	3.01	2.61
1 month (28 days)	1.53	2.31	2.21
2 months (56 days)	1.42	2.14	2.05
**FWB**			
Baseline	1.49	2.24	1.94
1 month (28 days)	2.25	3.37	3.24
2 months (56 days)	2.62	3.93	3.77
**Category**	**Criterion**		
	**1/3 SD**	**1/2SD**	**SEM**
**Group 2**			
**FACT-Hep**			
Baseline	5.25	7.88	6.87
1 month (28 days)	4.59	6.88	6.64
2 months (56 days)	0.5	0.75	5.80
**FACT-G**			
Baseline	4.31	6.47	5.64
1 month (28 days)	2.55	3.83	3.70
2 months (56 days)	1.64	2.46	1.82
**TOI**			
Baseline	3.58	5.38	4.69
1 month (28 days)	4.06	6.09	5.90
2 months (56 days)	1.82	2.74	5.95
**HepCS**			
Baseline	2.05	3.08	2.69
1 month (28 days)	2.35	3.52	3.4
2 months (56 days)	2.12	3.19	3.08
**PWB**			
Baseline	0.71	1.06	0.93
1 month (28 days)	1.24	2.01	1.96
2 months (56 days)	1.15	1.73	1.69
**SWB**			
Baseline	2.18	3.27	2.87
1 month (28 days)	1.29	1.94	1.86
2 months (56 days)	1.82	2.73	2.62
**EWB**			
Baseline	1.38	2.08	1.80
1 month (28 days)	1.54	2.31	2.21
2 months (56 days)	0.38	0.58	0.55
**FWB**			
Baseline	1.98	2.98	2.58
1 month (28 days)	2.11	3.17	3.04
2 months (56 days)	1.20	1.80	1.72
**Category**	**Criterion**		
	**1/3 SD**	**1/2SD**	**SEM**
**Group 3**			
**FACT-Hep**			
Baseline	4.99	7.49	6.53
1 month (28 days)	4.28	6.42	6.19
2 months (56 days)	7.78	11.66	11.24
**FACT-G**			
Baseline	2.58	3.87	3.37
1 month (28 days)	3.02	4.53	4.36
2 months (56 days)	4.94	7.42	7.15
**TOI**			
Baseline	4.61	6.92	6.04
1 month (28 days)	2.87	4.30	4.16
2 months (56 days)	4.47	6.71	6.50
**HepCS**			
Baseline	2.80	4.21	3.68
1 month (28 days)	2.30	3.46	3.34
2 months (56 days)	2.8	4.24	4.09
**PWB**			
Baseline	1.58	2.38	2.08
1 month (28 days)	1.84	2.76	2.70
2 months (56 days)	0.23	0.35	0.34
**SWB**			
Baseline	1.53	2.30	2.01
1 month (28 days)	2.30	3.46	2.49
2 months (56 days)	1.88	2.88	2.70
**EWB**			
Baseline	1.65	2.48	2.15
1 month (28 days)	2.17	3.26	3.13
2 months (56 days)	–	–	–
**FWB**			
Baseline	1.95	2.93	2.54
1 month (28 days)	1.74	2.61	2.52
2 months (56 days)	3.29	4.95	4.75

### ECOG performance

FACT-HEP data’s ability at baseline to correlate with clinical scores was evaluated based on ECOG performance, which was stratified into two levels, 0–1 and 2. For HepCS, a significant difference was found between patients with ECOG <2 and ≥2 (mean differences: 4.85, p=0.04). None of the other subcategories showed significant differences (S2 Table).

### Clinical responses

On day 28 of follow-up in Group 1, a significant difference in FWB scores was observed between patients with progressive and non-progressive diseases (mean difference = -11.11, p = 0.03). However, no other FACT-Hep subscales showed significant differences between these groups. Similarly, no significant differences were found in Groups 2 and 3. When data from Groups 1 and 2 (treated groups) were combined, the FWB subscale demonstrated a significant difference between progressive and non-progressive disease on day 28 of follow-up. Unfortunately, due to insufficient patients available for evaluation at days 56, 90, and 120 of follow-up, the association with disease progression could not be assessed. The mean (95% CI) or median (IQR) values for patients with progressive and non-progressive diseases are presented in S3 Table. Beyond differences in FACT-Hep and its subscale scores between progressive and non-progressive disease, no statistical differences were observed between survivors and non-survivors across all groups.

On day 56 of follow-up, significant differences in FACT-Hep (p = 0.022, mean difference = 28.9), TOI (p = 0.010, mean difference = 23.9), HepCS (p = 0.049, mean difference = 13.2), FWB (p = 0.024, mean difference = 6.9), and EWB (p = 0.046, mean difference = 8.20) scores were identified between survivors and non-survivors in the treated groups (Groups 1 and 2) compared with the untreated group (Group 3). These results are detailed in S4 Table.

### The clinical meaningfulness of treatment

Patients in Groups 1 and 2 showed a significant improvement in FACT-Hep (HRQOL) compared to baseline at 1 month (p=0.03) and 2 months (p=0.034), respectively. Improvement in PWB was observed in the treated groups (Group 1 and Group 2) at 1 month, while no significant difference in PWB was found in Group 3 (supportive care). These results align with clinical outcomes.

In comparison between groups, the Disease Control Rate (DCR) was significantly higher in Group 2 [9 (56.25%) cases] compared with Group 1 [5 (31.25%) cases, p<0.001] and Group 3 [5 (31.25%), p<0.001]. The Objective Response Rate (ORR) was significantly higher in Group 2 [3 (16.67%)] compared with Group 1 [0 (0%), p<0.001] and Group 3 [0 (0%), p<0.001]. Twelve patients in Group 3 died, while only 3 patients in Groups 1 and 2 died.

Significant differences in Progression-Free Survival (PFS), Overall Survival (OS), and Overall Survival Rate (OSR) were observed in Group 2 compared with Group 3. The proportion of patients with decreased or stable tumor progression was 37.5%, 62.5%, and 25% for Groups 1, 2, and 3, respectively. A significant reduction in the risk of tumor progression (Hazard Ratio [HR]: 0.065 [0.0042–0.61], p=0.034) was observed in Group 2. In addition, the risk of metastasis was significantly lower in the treated groups (Groups 1 and 2) compared with the control group (Group 3) (HR: 0.077 [0.0039–0.54], p=0.024) [33].

### Validity of FACT-Hep questionnaires to predict progression-free survival

For Group 1, significant difference between PFS and FWB scores (r=0.718, p=0.019) at baseline was found. No significant differences were found between PFS and other FACT-Hep subscales. For Group 2, the correlation between FACT-Hep (r-0.703, p=0.016), HepCS (r=-0.853, p<0.001), TOI (r=-0.745, p=0.009) and PFS at baseline were found. For Group 3, no significant correlation between FACT-Hep and PFS at baseline was found. On day 28, a significant positive correlation between EWB (differences in scores between baseline and follow-up) in Group 1 (r=0.757, p=0.049), a significant negative correlation between PWB in Group 2 (r=-0.809, p=0.015) and PFS were found. No significant correlation between FACT-Hep scores and PFS was found in Group 3. This may be due to the relatively small number of patients in Group 3.

### The assessment and management of adverse events

The NIH/NCL Common Toxicity Criteria (CTC) Grading System (version 4.03) was used to assess the adverse events associated with CMC-AL. The occurrence, pattern, intensity, and severity of adverse events were monitored throughout the study period. Adverse events likely related to CMC-AL were evaluated using the Naranjo algorithm. Physical examinations and vital sign monitoring were conducted weekly during the first three months and again at four months. Hematology, serum biochemistry, and urinalysis were assessed at 1, 2, 3, and 4 months. After evaluating the adverse events, it was found that four survivors in the treated group experienced adverse events, including ascites, chills, fever, nausea, vomiting, and loss of appetite. These adverse events were manageable according to standard guidelines.

## Discussions

### FACT-Hep scale structure: consistency and reliability

This study is the first to validate the applicability of the Thai version of the FACT-Hep scale in assessing health-related quality of life (HRQOL) in patients with advanced-stage intrahepatic cholangiocarcinoma (iCCA). It specifically evaluates its reliability, construct validity, Minimal Clinically Important Differences (MCIDs), and effect sizes. The assessment was conducted according to the FACIT.org guidelines. In general, the FACT-Hep, including its components (e.g., FACT-G, TOI, HepCS, PWB, SWB, EWB, and FWB), was shown to be a valid tool for evaluating HRQOL in patients with advanced-stage iCCA in this phase 2A study. The Cronbach’s alpha coefficients for all items exceeded the threshold of 0.7 (with an average of 0.8) at baseline, and again on days 28 (1 month) and 56 (2 months) after treatment. This indicates its reliability and robustness, supporting its role in guiding physicians in disease management. The Cronbach’s alpha coefficients of the QLQ-BIL21 at the baseline and follow-up time points were comparable with FACT-Hep. However, the coefficients at the 90-day (3 months) and 120-day (4 months) follow-ups were below 0.7, suggesting a moderate correlation with FACT-Hep. The relatively low coefficients during these periods may be attributed to the small sample size, indicating the need for further studies to confirm the reliability of FACT-Hep for HRQOL assessment in patients with advanced-stage iCCA.

In comparison with the FACT-Hep subscales (FACT-G, TOI, HepCS, and PWB), lower ICC values (<0.5) were observed for the test-retest from baseline to 3 months. This suggests moderate reliability (ICC > 0.4) for these subscales. Notably, when compared to QLQ-BIL21, the ICC values for all FACT-Hep subscales were higher than 0.9, indicating a high reliability of QLQ-BIL21 in assessing HRQOL in patients with CCA and gallbladder cancer. However, the higher ICC values for QLQ-BIL21 compared to FACT-Hep may be due to differences in the sample sizes of the two studies. The QLQ-BIL21 study involved patients with both CCA and gallbladder cancers, while this study focused solely on patients with advanced-stage iCCA. The type of cancer may act as a confounding factor in assessing HRQOL across different patient populations. Additionally, FACT-Hep might be better suited for assessing hepatocellular carcinoma (HCC) than advanced-stage iCCA, particularly in the HepCS subscale (ICC < 0.5). To further verify the applicability of FACT-Hep in CCA, additional studies are required to evaluate its construct validity and discriminant validity.

### Construct validity

Pearson’s correlation coefficients for most items in FACT-Hep were higher than 0.4 [32], indicating that these items were appropriately categorized within their respective subscales. However, three items—GP3, GF3, and HEP5—had Pearson’s r values below 0.4, suggesting potential misplacement within their subscale categories. For example, the item “HEP5: I have had a change in the way food tastes” may not fit well within the HepCS subscale. Although a change in taste can be linked to malnutrition, this issue is not specific to the hepatobiliary system. Therefore, HEP5 might be better placed under other subcategories, such as PWB (Physical Well-Being), as it addresses a broader, non-hepatic symptom.

Regarding discriminant validity, the total number of comparisons for FACT-Hep was 2,025, with 876 violation comparisons identified. According to Campbell and Fiske’s criteria for discriminant validity [35], the number of violations should be less than half of the total comparisons. Given this, the discriminant validity of FACT-Hep can be considered reliable.

When analyzing subscales individually, the number of violations for all categories, except HepCS, was below half of the total comparisons, indicating proper item placement within those subscales. However, the higher number of violations and the Pearson’s r value below 0.4 for HEP5 in the HepCS subscale suggest that many items in the HepCS subscale might correlate more strongly with other categories, such as FWB (Functional Well-Being) and PWB. Additionally, the violation of divergent (discriminant) validity in the HepCS subscale suggests that it is not entirely distinct from other subscales. Given that many HepCS items appear to align better with symptoms in the PWB category (e.g., ‘I have nausea,’ ‘I have a lack of energy,’ and ‘I have pain’), it would be appropriate to move these questions to the ‘Physical Well-Being’ subscale.

In contrast, all items in QLQ-BIL21 showed Pearson’s correlation coefficients greater than 0.4, indicating appropriate categorization, unlike FACT-Hep, where some items appear miscategorized [32]. This finding reinforces the reliability of QLQ-BIL21 for assessing HRQOL in this patient population.

Based on convergent and divergent validity, QLQ-BIL21 and QLQ-B30 are reliable tools for assessing HRQOL in patients with advanced-stage iCCA. However, further validation is needed for these tools, particularly in the specific context of iCCA patients, as the previous study [32] included patients with both CCA and gallbladder cancer. In addition to the quality of FACT-Hep validation, further evaluation of the sample-based quantity of FACT-Hep in CCA patients—considering both baseline and follow-up periods—is essential to ensure sufficient sample size. Therefore, analyzing the effect size in this study is also crucial to guarantee the robustness of the findings.

### Sensitivity to change and effect size of FACT-Hep over time

Overall, the changes in FACT-Hep scores were consistent with the observed effect sizes. Significant differences between baseline and follow-up scores were noted when effect sizes were large. For instance, significant decreases in HRQOL, with large effect sizes (>0.8), were observed from baseline to day 90 (3-month follow-up). In Group 1 patients (treated group), the decrease in HRQOL at the 3-month follow-up for the FACT-G and FWB subscales is likely attributable to adverse effects related to AL therapy. Similarly, in Group 2 patients (treated group), significant decreases in FACT-Hep, TOI, and HepCS subscales were observed after 28 days (1 month) of treatment.

Furthermore, subscales with large effect sizes following the 2- and 3-month follow-ups demonstrated significant differences between baseline and follow-up scores. In contrast, no significant differences were found in the control group (Group 3). These decreases in FACT-Hep scores are consistent with the gastrointestinal adverse effects of CMC-AL reported in the phase 2A clinical trial [33].

For the 1-, 2-, and 4-month follow-ups (28, 56, and 120 days) in Group 1, effect sizes ranged from low to moderate, with no significant differences observed. These findings suggest that larger effect sizes increase the likelihood of detecting significant differences [36].

In evaluating the quality of FACT-Hep validation and the number of samples from patients with cholangiocarcinoma (CCA) treated with AL, we also assessed the patients’ performance using the ECOG score. This evaluation determined whether the patients met the criteria for receiving AL therapy in the Phase IIA clinical trial, as outlined in the protocol. The ECOG score is widely used to assess cancer patients’ performance status prior to receiving chemotherapy. Further clinical evaluation of the correlation between FACT-Hep and the ECOG score would be valuable in understanding how changes in the ECOG score relate to changes in quality of life (QoL).

### ECOG performance

The FACT-Hep questionnaires, including FACT-Hep, FACT-G, TOI, HepCS, FWB, SWB, EWB, and PWB, have demonstrated the ability to assess the clinical performance of CMC-AL treatment following ECOG stratification. The HepCS domain, which covers signs and symptoms relevant to patients with advanced-stage iCCA, such as tiredness, jaundice, adverse effects, and weight loss (similar to the EORTC QLQ-BIL21), showed a significant decrease in scores for patients with an ECOG score of 2. This suggests that patients with advanced-stage iCCA, who are classified with an ECOG of 2, experience a worse clinical performance than those with an ECOG of 1.

Additionally, the FWB domain assesses functional ability in daily life, which correlates with the ECOG performance score. However, both groups of patients with an ECOG of 1 and 2 exhibited restricted FWB, such as limited physical activity and difficulties in performing housework. As a result, these criteria may not be sensitive enough to detect significant differences between the two groups. This observation may also explain the lack of a significant difference in PWB for patients with ECOG scores of 1 and 2.

When compared with the EORTC QLQ-BIL21, no significant differences were observed in FWB (e.g., psychological assessments or tiredness) between patients with an ECOG of 1 and 2 [32]. However, a significant difference in the tiredness subscale was noted between patients with KPS ≥70 and <70 in the EORTC QLQ-BIL21 assessment [32]. This finding suggests that the ECOG performance scale may have lower discriminant properties than the KPS scale in distinguishing between two patient groups. Similarly, this pattern was observed in the evaluation of anxiety (EWB) in the EORTC QLQ-BIL21 questionnaire.

Furthermore, in addition to EWB and SWB, FWB also showed significant differences in the assessment of tiredness in the EORTC QLQ-BIL21 and KPS performance. However, there were no significant differences between FACT-Hep and ECOG performance for patients with ECOG scores of 1 and 2. FACT-Hep, in contrast, provides a more comprehensive assessment of emotional, psychological, and functional well-being (EWB, PWB, and FWB) than the EORTC QLQ-BIL21 [32].

Further clinical evaluation of ECOG performance in patients with ECOG scores ≤2 and >2 is needed to enhance the ability of the ECOG performance scale to detect correlations between clinical status and HRQOL in patients with advanced-stage iCCA. Moreover, evaluating the applicability of FACT-Hep in detecting changes in HRQOL scores and subscales as a clinical response to AL therapy in CCA patients is crucial for refining its role in clinical assessments.

### Clinical responses

The significant decrease in FWB scores after one month (28 days) of treatment in patients with progressive disease, compared to those with non-progressive disease, may reflect the limited functional well-being (FWB) following CMC-AL therapy. Interestingly, patients with non-progressive disease exhibited higher FWB scores [18] than those with progressive disease [12]. This suggests that CMC-AL may improve FWB in certain patients, potentially enhancing their ability to perform daily activities, including housework, and improve sleep quality. However, other subscales of FACT-Hep did not show significant differences between the progressive and non-progressive disease groups. Both groups experienced similar adverse effects from CMC-AL therapy, which may have similarly impacted their physical well-being (PWB), social well-being (SWB), and emotional well-being (EWB). These adverse effects could explain the lack of significant differences observed in the remaining FACT-Hep subscales. Additionally, the limited number of patients in each group may have reduced the statistical power to detect subtle differences between responders (non-progressive disease) and non-responders (progressive disease).

In the untreated group, no significant differences were observed in HepCS subscale scores for hepatobiliary tract cancers, including intrahepatic cholangiocarcinoma (iCCA). This may be because the HepCS subscale lacks specificity for iCCA. Developing tools that focus on the unique signs and symptoms of iCCA—such as jaundice, fatigue, limited ability to perform housework, and itching—could improve the sensitivity of the assessment.

When compared to the EORTC QLQ-BIL21, which tracks clinical changes over time, FACT-Hep provides more specific questions (HepCS) related to cholangiocarcinoma (CCA). However, QLQ-BIL21 focuses on clinical responses based on criteria such as RECIST or modified RECIST [32]. To date, no study has specifically investigated changes in HRQOL scores, including those from EORTC QLQ-BIL21 and QLQ-B30, in relation to clinical response (progressive vs. non-progressive disease) [32]. Developing new tools to explore this relationship in iCCA, extrahepatic CCA (eCCA), and hilar CCA (HCCA) would be highly beneficial. These tools could predict clinical responses based on HRQOL changes, enabling physicians to use HRQOL as a non-invasive guide to inform treatment decisions.

Previous HRQOL analyses in Thailand using FACT-Hep revealed that a 9-point increase in FACT-Hep and HepCS scores was associated with significantly reducing mortality risk (HR: 0.56, 95% CI: 0.32–0.95) [31]. In this study, the combined treated groups (Groups 1 and 2) showed significant differences in FACT-Hep scores compared to the untreated group (Group 3). Notably, survivors in Group 3 had lower mean FACT-Hep scores than non-survivors. Conversely, higher FACT-Hep scores were associated with better prognosis in patients with pancreatic cancer [37]. These findings suggest that the adverse effects of CMC-AL therapy may influence FACT-Hep and its subscale scores.

While the EORTC QLQ-BIL21 covers aspects such as eating behavior (FWB), jaundice (as a marker of disease progression), tiredness (PWB), anxiety (EWB), pain (related to disease progression), treatment-related adverse effects, drainage, and weight loss (PWB), FACT-Hep provides more detailed symptom-specific questions (HepCS) relevant to CCA. Although EORTC QLQ-BIL21 monitors clinical changes, FACT-Hep’s more specific questions allow a deeper understanding of symptoms directly affecting HRQOL in patients with advanced-stage iCCA.

In conclusion, FACT-Hep’s benefits and reliability in assessing HRQOL in patients with advanced-stage iCCA are significant. This reinforces its value as an HRQOL assessment tool. Further evaluation of the relationship between FACT-Hep scores and clinical outcomes will be crucial for advancing its role in clinical practice.

### Correlation between FACT-Hep questionnaires and clinical outcomes

For Group 1 (treated group), a positive correlation between FWB and PFS scores at baseline suggests that patients with high FWB scores tend to have more favorable PFS than those with lower FWB scores. This highlights the potential importance of FWB in predicting treatment outcomes for these patients. In contrast, Group 2 exhibited a significant negative correlation between FACT-Hep, HepCS, TOI scores and PFS at baseline. This negative correlation may be attributed to the performance status of Group 2 patients, many of whom had an ECOG score of 2, indicating restricted physical and functional activity. Group 1 patients had a higher proportion with an ECOG score of 1, suggesting better overall functional capacity. As a result, the baseline FACT-Hep scores should not be relied upon as an independent predictor of PFS in patients with advanced-stage iCCA.

Furthermore, a significantly positive correlation between EWB in Group 1 and a negative correlation between PWB in Group 2 with PFS suggests that adverse effects related to CMC-AL may have contributed to the decline in PWB scores. Patients in Group 2 received a higher daily dose of CMC-AL, which could have caused more pronounced adverse effects, thereby influencing PWB scores. Interestingly, patients with lower PWB scores seemed to have improved PFS compared to those whose PWB scores remained unchanged or increased. Additionally, the positive correlation with EWB implies that improvements in emotional well-being may significantly enhance PFS in patients with advanced-stage iCCA.

This study primarily aims to validate FACT-Hep as an effective tool for assessing the quality of life in patients with intrahepatic cholangiocarcinoma (iCCA) following treatment by comparing baseline and follow-up measurements. The validity and reliability of FACT-Hep in detecting clinically meaningful changes in health status (HRQOL) are essential. Effect sizes are helpful in capturing changes before and after treatment within a group, providing a crucial understanding of treatment outcomes. However, this method may be limited in detecting subtle changes in health status and the treatment’s impact over multiple measurement points. A multivariate linear mixed model would likely provide a more robust statistical approach for evaluating the effects of treatment on the progression of longitudinal measurements.

A limitation of the current study is its relatively small sample size, which may have affected the ability to detect significant differences both within and between groups and in clinical responses. A larger clinical trial is recommended to better assess HRQOL using FACT-Hep in patients with cholangiocarcinoma (iCCA, eCCA, and hCCA). Additionally, considering the natural history of iCCA, the follow-up period of up to 4 months may be insufficient, as the average overall survival (OS) for Thai patients (from Northeastern Thailand) with advanced-stage iCCA is approximately 4 months [38]. Early-stage recruitment would provide patients with adequate survival time to complete the survey. It should also be noted that this study had limitations, including the reliance on clinical signs and symptoms combined with radiological examinations for diagnosing iCCA. All patients in the study refused chemotherapy, so tissue biopsies and histological examinations were not conducted to confirm the diagnosis of iCCA.

This study employed the FACT-Hep questionnaire to assess health-related quality of life (HRQOL) following AL therapy. However, its application to patients with intrahepatic cholangiocarcinoma (iCCA) may have limitations, as AL is not yet a standard treatment for this condition. Therefore, further clinical studies are needed to evaluate HRQOL in cholangiocarcinoma (biliary tract cancer) patients after first-line therapies and other herbal treatments, with extended follow-up periods of up to 12 months. Such research would help establish the validity and reliability of the FACT-Hep questionnaire in this patient population and provide a clearer understanding of treatment effects on HRQOL.

Gemcitabine-based chemotherapy is typically the preferred palliative treatment for inoperable iCCA. However, this regimen has demonstrated poor clinical outcomes and is often associated with unacceptable adverse events, especially in patients with stage IV disease. These negative outcomes could impact the validity of HRQOL assessments.

On a positive note, this study suggests that AL can serve as a surrogate marker for evaluating treatment impacts on quality of life (QoL), as evidenced by the established reliability and validity of the FACT-Hep tool. Additionally, the CMC-AL used in this study complies with Good Manufacturing Practice (GMP) standards, ensuring that the Active Pharmaceutical Ingredients (APIs) in each capsule are standardized. This further underscores the reliability of the herbal medicine used in the study, which complements Western chemotherapy treatments.

## Conclusion

The FACT-Hep has proven to be an effective tool for assessing HRQOL in patients with advanced-stage iCCA, supported by its strong reliability and validity metrics. At baseline and during the 1-, 2-, and 3-month follow-ups, the Cronbach’s alpha values for FACT-Hep and its subscales exceeded 0.8, demonstrating excellent internal consistency. As indicated by ICC values, test-retest reliability ranged from 0.427 to 0.795 (p < 0.001) over the same period, comparable to the reliability of QLQ-BIL21.

The construct validity of FACT-Hep was confirmed for most components, meeting the set criteria for both convergent and divergent (discriminant) validity. However, the HepCS subscale showed limitations, with more than half of its discriminant validity comparison violations, indicating areas requiring further refinement.

FACT-Hep demonstrated sensitivity to changes in HRQOL scores associated with clinical responses. In the treated group, FWB scores showed a significant difference between patients with progressive and non-progressive disease on day 28 (p = 0.03). Among survivors and non-survivors in the treated group, significant differences were observed in several domains: FACT-Hep (p = 0.002), TOI (p = 0.01), HepCS (p = 0.049), FWB (p = 0.024), and EWB (p = 0.046).

Further research involving larger cohorts of advanced-stage iCCA patients is recommended to validate the consistency, reliability, and broader applicability of FACT-Hep in clinical settings.

## Supporting information

S1 Table Cronbach’s alpha coefficient of FACT-Hep for each item at baseline, and on days 28 (1 month), 56 (2 months), 90 (3 months), and 120 (4 months) of treatment follow-up. (DOCX)

S2 Table Comparisons of FACT-Hep scores and its subscales between patients with ECOG of 1≤ and ECOG of 2. (DOCX)

S3 Table Comparison of FACT-hep and its subscales in iCCA patients with progressive and non-progressive disease on day 28 (1 month) and 56 (2 months) of treatment follow-up. (DOCX)

S4 Table Comparison of FACT-Hep and its subscales in survivors and non-survivors on day 28 (1 month) and 56 (2 months) of treatment follow-up. (DOCX)

S1 File Methods: Inclusion and exclusion criteria. (DOCX)

S2 File FACT-Hep 4 Thai version. (DOCX)

S3 File FACT-Hep 4 English version S4 Table Comparison of FACT-Hep and its subscales in survivors and non-survivors on day 28 (1 month) and 56 (2 months) of treatment follow-up. (DOCX)

## Abbreviation

DCS: disease control rate
EORTC QLQ-BIL21: European Organisation for Research and Treatment of Cancer Quality of Life Questionnaire-BIL21
EORTC QLQ-C30: European Organization for Research and Treatment of Cancer Quality of Life Questionnaire-Core 30
EWB: emotional well-being
FWB: functional well-being
FACT-Hep: Functional Assessment of Cancer Therapy Hepatobiliary
GCP: good clinical practice
HepCS: hepatobiliary cancer subscale
HRQOL: health-related quality of life
ICC: intraclass correlation coefficient
iCCA: Intrahepatic cholangiocarcinoma
IQR: interquartile range
MCID: minimal clinically important difference
OS: overall survival
QoL: quality of life
PFSR: Progression-free survival rate
PWB: Physical well-being
RECIST: Response Evaluation Criteria in Solid Tumours
SEM: standard error of the mean
SWB: social/family well-being
TOI: trial outcome index

## References

[1] Wohlleber K, Heger P, Probst P, Engel C, Diener MK, Mihaljevic AL. Health-related quality of life in primary hepatic cancer: a systematic review assessing the methodological properties of instruments and a meta-analysis comparing treatment strategies. Qual Life Res. 2021;30(9):2429–66. doi: 10.1007/s11136-021-02810-8 34283381 PMC8405513

[2] Bruix J, Qin S, Merle P, Granito A, Huang Y-H, Bodoky G, et al. Regorafenib for patients with hepatocellular carcinoma who progressed on sorafenib treatment (RESORCE): a randomised, double-blind, placebo-controlled, phase 3 trial. Lancet. 2017;389(10064):56–66. doi: 10.1016/S0140-6736(16)32453-9 27932229

[3] Cao W, Li J, Hu C, Shen J, Liu X, Xu Y, et al. Symptom clusters and symptom interference of HCC patients undergoing TACE: a cross-sectional study in China. Support Care Cancer. 2013;21(2):475–83. doi: 10.1007/s00520-012-1541-5 23010958

[4] Cebon J, Findlay M, Hargreaves C, Stockler M, Thompson P, Boyer M, et al. Somatostatin receptor expression, tumour response, and quality of life in patients with advanced hepatocellular carcinoma treated with long-acting octreotide. Br J Cancer. 2006;95(7):853–61. doi: 10.1038/sj.bjc.6603325 16953241 PMC2360532

[5] Chang-Chien W-Y, Lee K-T, Shi H-Y. P0132 A longitudinal prospective analysis of depression, anxiety, and quality of life in patients with hepatocellular carcinoma. Eur J Cancer. 2014;50:e46–7. doi: 10.1016/j.ejca.2014.03.176

[6] Chay WY, Tham CK, Toh HC, Lim HY, Tan CK, Lim C, et al. Coriolus versicolor (Yunzhi) use as therapy in advanced hepatocellular carcinoma patients with poor liver function or who are unfit for standard therapy. J Altern Complement Med. 2017;23(8):648–52. doi: 10.1089/acm.2016.0136 28375640

[7] Cheng A-L, Kang Y-K, Chen Z, Tsao C-J, Qin S, Kim JS, et al. Efficacy and safety of sorafenib in patients in the Asia-Pacific region with advanced hepatocellular carcinoma: a phase III randomised, double-blind, placebo-controlled trial. Lancet Oncol. 2009;10(1):25–34. doi: 10.1016/S1470-2045(08)70285-7 19095497

[8] Chiu C-C, Lee K-T, Wang J-J, Sun D-P, Lee H-H, Shi H-Y. Health-related quality of life before and after surgical resection of hepatocellular carcinoma: a prospective study. Asian Pac J Cancer Prev. 2018;19(1):65–72. doi: 10.22034/APJCP.2018.19.1.65 29373894 PMC5844638

[9] Dollinger MM, Lautenschlaeger C, Lesske J, Tannapfel A, Wagner A-D, Schoppmeyer K, et al. Thymostimulin versus placebo for palliative treatment of locally advanced or metastasised hepatocellular carcinoma: a phase III clinical trial. BMC Cancer. 2010;10:457. doi: 10.1186/1471-2407-10-457 20735834 PMC2936330

[10] Gmür A, Kolly P, Knöpfli M, Dufour J-F. FACT-Hep increases the accuracy of survival prediction in HCC patients when added to ECOG performance status. Liver Int. 2018;38(8):1468–74. doi: 10.1111/liv.13711 29389088

[11] Kolligs FT, Bilbao JI, Jakobs T, Iñarrairaegui M, Nagel JM, Rodriguez M, et al. Pilot randomized trial of selective internal radiation therapy vs. chemoembolization in unresectable hepatocellular carcinoma. Liver Int. 2015;35(6):1715–21. doi: 10.1111/liv.12750 25443863

[12] Liu J, Wang Y, Zhang D, Liu B, Ou Q. Comparison of survival and quality of life of hepatectomy and thrombectomy using total hepatic vascular exclusion and chemotherapy alone in patients with hepatocellular carcinoma and tumor thrombi in the inferior vena cava and hepatic vein. Eur J Gastroenterol Hepatol. 2012;24(2):186–94. doi: 10.1097/MEG.0b013e32834dda64 22081008

[13] Nowak AK, Cebon J, Hargreaves C, Dhillon H, Findlay M, Gebski V, et al. Assessment of health-related quality of life and patient benefit as outcome measures for clinical trials in hepatocellular carcinoma. Asia-Pac J Clncl Oncology. 2008;4(1):55–67. doi: 10.1111/j.1743-7563.2008.00142.x

[14] Qiao C-X, Zhai X-F, Ling C-Q, Lang Q-B, Dong H-J, Liu Q, et al. Health-related quality of life evaluated by tumor node metastasis staging system in patients with hepatocellular carcinoma. World J Gastroenterol. 2012;18(21):2689–94. doi: 10.3748/wjg.v18.i21.2689 22690079 PMC3370007

[15] Ryu E, Kim K, Cho MS, Kwon IG, Kim HS, Fu MR. Symptom clusters and quality of life in Korean patients with hepatocellular carcinoma. Cancer Nurs. 2010;33(1):3–10. doi: 10.1097/NCC.0b013e3181b4367e 19926981

[16] Salem R, Gilbertsen M, Butt Z, Memon K, Vouche M, Hickey R, et al. Increased quality of life among hepatocellular carcinoma patients treated with radioembolization, compared with chemoembolization. Clin Gastroenterol Hepatol. 2013;11(10):1358-1365.e1. doi: 10.1016/j.cgh.2013.04.028 23644386

[17] Somjaivong B, Thanasilp S, Preechawong S, Sloan R. The influence of symptoms, social support, uncertainty, and coping on health-related quality of life among cholangiocarcinoma patients in northeast Thailand. Cancer Nurs. 2011;34(6):434–42. doi: 10.1097/NCC.0b013e31820d0c3f 21372698

[18] Steel J, Baum A, Carr B. Quality of life in patients diagnosed with primary hepatocellular carcinoma: hepatic arterial infusion of Cisplatin versus 90-Yttrium microspheres (Therasphere). Psychooncology. 2004;13(2):73–9. 14872525 10.1002/pon.725

[19] Steel J, Hess SA, Tunke L, Chopra K, Carr BI. Sexual functioning in patients with hepatocellular carcinoma. Cancer. 2005;104(10):2234–43. doi: 10.1002/cncr.21450 16220558

[20] Steel JL, Geller DA, Carr BI. Proxy ratings of health related quality of life in patients with hepatocellular carcinoma. Qual Life Res. 2005;14(4):1025–33. doi: 10.1007/s11136-004-3267-4 16041898

[21] Steel JL, Chopra K, Olek MC, Carr BI. Health-related quality of life: hepatocellular carcinoma, chronic liver disease, and the general population. Qual Life Res. 2007;16(2):203–15. doi: 10.1007/s11136-006-9111-2 17119847

[22] Steel JL, Geller DA, Robinson TL, Savkova AY, Brower DS, Marsh JW, et al. Health-related quality of life as a prognostic factor in patients with advanced cancer. Cancer. 2014;120(23):3717–21. doi: 10.1002/cncr.28902 25104581 PMC4239171

[23] Toro A, Pulvirenti E, Palermo F, Di Carlo I. Health-related quality of life in patients with hepatocellular carcinoma after hepatic resection, transcatheter arterial chemoembolization, radiofrequency ablation or no treatment. Surg Oncol. 2012;21(1):e23-30. doi: 10.1016/j.suronc.2011.10.005 22104002

[24] Wang L, Wang Y, Tang L, Feng C, Liu X, Zhang R, et al. Quality of life and the relevant factors in patients with chronic hepatitis B. Hepatogastroenterology. 2012;59(116):1036–42. doi: 10.5754/hge11867 22328288

[25] Woradet S, Promthet S, Songserm N, Parkin DM. Factors affecting health-related quality of life in patients with cholangiocarcinoma in the northeastern region of Thailand. Cancer Nurs. 2015;38(6):E46-51. doi: 10.1097/NCC.0000000000000233 25785579

[26] Zhang Y, Fan W, Zhu K, Lu L, Fu S, Huang J, et al. Sorafenib continuation or discontinuation in patients with unresectable hepatocellular carcinoma after a complete response. Oncotarget. 2015;6(27):24550–9. doi: 10.18632/oncotarget.4076 26093084 PMC4695205

[27] Zheng W, Wu J, Xiao J-R, Guo Q. Survival and health-related quality of life in patients with spinal metastases originated from primary hepatocellular carcinoma. J Evid Based Med. 2013;6(2):81–9. doi: 10.1111/jebm.12034 23829800

[28] Mihalache F, Tantau M, Diaconu B, Acalovschi M. Survival and quality of life of cholangiocarcinoma patients: a prospective study over a 4 year period. J Gastrointestin Liver Dis. 2010;19(3):285–90. 20922193

[29] Ortner MEJ, Caca K, Berr F, Liebetruth J, Mansmann U, Huster D, et al. Successful photodynamic therapy for nonresectable cholangiocarcinoma: a randomized prospective study. Gastroenterology. 2003;125(5):1355–63. doi: 10.1016/j.gastro.2003.07.015 14598251

[30] Park DH, Lee SS, Park SE, Lee JL, Choi JH, Choi HJ, et al. Randomised phase II trial of photodynamic therapy plus oral fluoropyrimidine, S-1, versus photodynamic therapy alone for unresectable hilar cholangiocarcinoma. Eur J Cancer. 2014;50(7):1259–68. doi: 10.1016/j.ejca.2014.01.008 24485665

[31] Woradet S, Songserm N, Promthet S, Parkin DM. Health-related quality of life and survival of cholangiocarcinoma patients in northeastern region of Thailand. PLoS One. 2016;11(9):e0163448. doi: 10.1371/journal.pone.0163448 27685448 PMC5042427

[32] Kaupp-Roberts SD, Yadegarfar G, Friend E, O’Donnell CM, Valle JW, Byrne C, et al. Validation of the EORTC QLQ-BIL21 questionnaire for measuring quality of life in patients with cholangiocarcinoma and cancer of the gallbladder. Br J Cancer. 2016;115(9):1032–8. doi: 10.1038/bjc.2016.284 27673364 PMC5117782

[33] Na-Bangchang K., Tongsiri N., Plengsuriyakarn T., Saeheng T., Kongjam P., Kulma I., et al. Phase IIa clinical trial to evaluate safety and efficacy of capsule formulation of the standardized extract of Atractylodes Lancea (Thunb) DC in patients with advanced-stage intrahepatic cholangiocarcinoma. World J Chin Med. 2024. 10.1177/15347354231223967 PMC10832411 38291969

[34] Steel JL, Eton DT, Cella D, Olek MC, Carr BI. Clinically meaningful changes in health-related quality of life in patients diagnosed with hepatobiliary carcinoma. Ann Oncol. 2006;17(2):304–12. doi: 10.1093/annonc/mdj072 16357021

[35] CAMPBELL DT, FISKE DW. Convergent and discriminant validation by the multitrait-multimethod matrix. Psychol Bull. 1959;56(2):81–105. 13634291

[36] Kazis LE, Anderson JJ, Meenan RF. Effect sizes for interpreting changes in health status. Med Care. 1989;27(3 Suppl):S178-89. doi: 10.1097/00005650-198903001-00015 2646488

[37] Cella D, Butt Z, Kindler HL, Fuchs CS, Bray S, Barlev A, et al. Validity of the FACT Hepatobiliary (FACT-Hep) questionnaire for assessing disease-related symptoms and health-related quality of life in patients with metastatic pancreatic cancer. Qual Life Res. 2013;22(5):1105–12. doi: 10.1007/s11136-012-0217-4 22678353

[38] Luvira V, Nilprapha K, Bhudhisawasdi V, Pugkhem A, Chamadol N, Kamsa-ard S. Cholangiocarcinoma patient outcome in Northeastern Thailand: single-center prospective study. Asian Pac J Cancer Prev. 2016;17(1):401–6. doi: 10.7314/apjcp.2016.17.1.401 26838246

