# Supplementary material for: The first validation of the Functional Assessment of Cancer Therapy Hepatobiliary (FACT-Hep) for evaluating health-related quality of life (HRQOL) in patients with advanced-stage intrahepatic cholangiocarcinoma (biliary tract cancer)
Source: PLoS One. 2025 Apr 28;20(4):e0321618. doi: 10.1371/journal.pone.0321618 (PMC12036939; doi:10.1371/journal.pone.0321618)
Supplement: S1 Table — (DOCX) [file pone.0321618.s001.docx]

**Table S1. Cronbach’s alpha coefficient of FACT-Hep for each item at baseline, and on days 28 (1 month), 56 (2 months), 90 (3 months), and 120 (4 months) of treatment follow-up.**

| **Item** | **Baseline**  **(Day 1)** | **1 month**  **( Day 28)** | **2 months**  **(Day 56)** | **3 months**  **(Day 90)** | **4 months**  **(Day 120)** |
| --- | --- | --- | --- | --- | --- |
| **GP1** | 0.809 | 0.762 | 0.769 | 0.200 | 0.519 |
| **GP2** | 0.811 | 0.763 | 0.712 | 0.298 | 0.541 |
| **GP3** | 0.81 | 0.770 | 0.766 | 0.180 | 0.470 |
| **GP4** | 0.8 | 0.749 | 0.736 | 0.278 | 0.520 |
| **GP5** | 0.814 | 0.758 | 0.758 | 0.235 | 0.541 |
| **GP6** | 0.804 | 0.759 | 0.732 | 0.143 | 0.515 |
| **GP7** | 0.808 | 0.766 | 0.754 | 0.193 | 0.563 |
| **GS1** | 0.807 | 0.772 | 0.748 | 0.362 | 0.590 |
| **GS2** | 0.803 | 0.771 | 0.749 | 0.337 | 0.569 |
| **GS3** | 0.807 | 0.770 | 0.747 | 0.281 | 0.590 |
| **GS4** | 0.806 | 0.766 | 0.752 | 0.277 | 0.549 |
| **GS5** | 0.808 | 0.765 | 0.752 | 0.257 | 0.599 |
| **GS6** | 0.818 | 0.782 | 0.752 | 0.455 | 0.598 |
| **GS7** | 0.813 | 0.771 | 0.748 | 0.258 | 0.562 |
| **GE1** | 0.815 | 0.774 | 0.748 | 0.291 | 0.519 |
| **GE2** | 0.804 | 0.761 | 0.750 | 0.370 | 0.600 |
| **GE3** | 0.814 | 0.772 | 0.739 | 0.294 | 0.517 |
| **GE4** | 0.818 | 0.771 | 0.729 | 0.261 | 0.517 |
| **GE5** | 0.817 | 0.769 | 0.743 | 0.242 | 0.532 |
| **GE6** | 0.812 | 0.772 | 0.732 | 0.166 | 0.529 |
| **GF1** | 0.817 | 0.772 | 0.751 | 0.429 | 0.576 |
| **GF2** | 0.822 | 0.770 | 0.746 | 0.403 | 0.608 |
| **GF3** | 0.81 | 0.766 | 0.748 | 0.255 | 0.604 |
| **GF4** | 0.803 | 0.758 | 0.749 | 0.247 | 0.550 |
| **GF5** | 0.813 | 0.782 | 0.779 | 0.301 | 0.597 |
| **GF6** | 0.81 | 0.766 | 0.746 | 0.317 | 0.600 |
| **GF7** | 0.809 | 0.770 | 0.750 | 0.314 | 0.560 |
| **C1** | 0.804 | 0.761 | 0.752 | 0.178 | 0.525 |
| **C2** | 0.8 | 0.761 | 0.735 | 0.263 | 0.486 |
| **C3** | 0.813 | 0.768 | 0.750 | 0.239 | 0.592 |
| **C4** | 0.812 | 0.765 | 0.768 | 0.317 | 0.579 |
| **C5** | 0.814 | 0.774 | 0.762 | 0.239 | 0.558 |
| **C6** | 0.811 | 0.772 | 0.762 | 0.412 | 0.599 |
| **HEP1** | 0.808 | 0.770 | 0.763 | 0.210 | 0.505 |
| **CNS7** | 0.813 | 0.763 | 0.744 | 0.285 | 0.510 |
| **CX6** | 0.814 | 0.778 | 0.730 | 0.247 | 0.524 |
| **H17** | 0.802 | 0.762 | 0.751 | 0.318 | 0.519 |
| **AN7** | 0.814 | 0.774 | 0.754 | 0.341 | 0.619 |
| **HEP2** | 0.808 | 0.778 | 0.721 | 0.200 | 0.535 |
| **HEP3** | 0.813 | 0.775 | 0.763 | 0.290 | 0.538 |
| **HEP4** | 0.813 | 0.771 | 0.746 | 0.252 | 0.515 |
| **HEP5** | 0.812 | 0.767 | 0.754 | 0.211 | 0.486 |
| **HEP6** | 0.81 | 0.772 | 0.728 | 0.228 | 0.551 |
| **HN2** | 0.803 | 0.755 | 0.737 | 0.123 | 0.500 |
| **HEP8** | 0.804 | 0.748 | 0.769 | 0.184 | 0.519 |
