# Supplementary material for: The first validation of the Functional Assessment of Cancer Therapy Hepatobiliary (FACT-Hep) for evaluating health-related quality of life (HRQOL) in patients with advanced-stage intrahepatic cholangiocarcinoma (biliary tract cancer)
Source: PLoS One. 2025 Apr 28;20(4):e0321618. doi: 10.1371/journal.pone.0321618 (PMC12036939; doi:10.1371/journal.pone.0321618)
Supplement: S2 Table — (DOCX) [file pone.0321618.s002.docx]

**Table S2. Comparisons of FACT-Hep scores and its subscales between patients with ECOG of 1≤ and ECOG of 2.**

| **Categories** | **Mean difference** | **Standard errors** | **Mean difference (95% CI)** | | **t/ U** | **df** | **p-value** |
| --- | --- | --- | --- | --- | --- | --- | --- |
|  |  |  | **Lower bound** | **Upper bound** |  |  |  |
| **FACT-Hep**** | - | 35.77 | - | - | 237.5 | - | 0.16 |
| **FACT-G**** | - | 35.77 | - | - | 206.5 |  | 0.6 |
| **TOI** | -7.64 | 4.64 | -17.03 | 1.75 | -1.647 | 38 | 0.108 |
| **HepCS** | -5.31 | 2.57 | -10.51 | -0.103 | -2.064 | 38 | 0.046* |
| **FWB** | 0.12 | 1.84 | -3.61 | 3.85 | 0.65 | 38 | 0.474 |
| **EWB**** | - | 35.53 | - | - | 206.5 | - | 0.6 |
| **SWB**** | - | 35.28 | - | - | 175 | - | 0.74 |
| **PWB**** | - | 35.65 | - | - | 226 | - | 0.292 |

**Statistical significant difference**Mann-Whitney U test*
