# Supplementary material for: The first validation of the Functional Assessment of Cancer Therapy Hepatobiliary (FACT-Hep) for evaluating health-related quality of life (HRQOL) in patients with advanced-stage intrahepatic cholangiocarcinoma (biliary tract cancer)
Source: PLoS One. 2025 Apr 28;20(4):e0321618. doi: 10.1371/journal.pone.0321618 (PMC12036939; doi:10.1371/journal.pone.0321618)
Supplement: S3 Table — (DOCX) [file pone.0321618.s003.docx]

**Table S3 Comparison of FACT-hep and its subscales in iCCA patients with progressive and non-progressive disease on day 28 (1 month) and 56 (2 months) of treatment follow-up.**

| **Category** | **Progressive disease** | | **Non-progressive disease** | |
| --- | --- | --- | --- | --- |
|  | **1 month (28 days)** | **2 months (56 days)** | **1 month (28 days)** | **2 months (56 days)** |
| **Group 1** | | | | |
| **FACT-Hep** | 66.4 (48.56-84.23) | 73.5 (67.14-79.85) | 70.5 (-12.09-153.09) | -^a^ |
| **FACT-G** | 51.6 (35.06-68.13) | 50 (-0.82-100.82) | 54 (-60-168.35) | -^a^ |
| **TOI** | 35.4 (24.95-45.84) | 49 (-1.82-99.82) | 43 (4.88-81.11) | -^a^ |
| **HepCS** | 17 (10.19-23.80) | 23.5 (-33.67-80.67) | 16.5 (-15.26-48.26) | -^a^ |
| **PWB** | 8 (2.65-13.34) | 4 (-34.11-42.11) | 5 (-7.70-17.70) | -^a^ |
| **SWB** | 28 (13.5) | 20.5 (-36.67-77.67) | 24 (-14.11-62.11) | -^a^ |
| **EWB** | 10 (7) | 4 (-8.70-16.70) | 3.5 (-2.85-9.85) | -^a^ |
| **FWB** | 10.4 (5.70-15.09) | 21.5 (-22.97-65.97) | 21.5 (-35.67-78.67) | -^a^ |
| **Group 2** | | | | |
| **FACT-Hep** | 60.5 (-149.15-270.15) | -^a^ | 74.33(63.86-84.79) | 63.5 (44.44-82.55) |
| **FACT-G** | 45 (-82.06-172.06) | -^a^ | 54.16 (49.79-58.54) | 50.5 (18.73-82.26) |
| **TOI** | 35.5 (-123.32-194.32) | -^a^ | 43.5 (31.70-55.29) | 35 (9.58-60.41) |
| **HepCS** | 15.5 (-67.09-98.09) | -^a^ | 20.16 (13.03-27.29) | 13 (0.29-25.70) |
| **PWB** | 4 (-46.82-54.82) | -^a^ | 5.67 (1.53-9.79) | 1.5 (-17.55-20.55) |
| **SWB** | 21.5 (15.14-27.85) | -^a^ | 24.33 (19.79-28.86) | 26.5 (7.44-45.55) |
| **EWB** | 3.5 (-40.97-47.97) | -^a^ | 6.5 (1.54-11.45) | 2 (-23.41-27.41) |
| **FWB** | 16 (-9.41-41.41) | -^a^ | 17.67 (9.96-25.36) | 20.5 (-11.26-52.26) |
| **Group 3** | | | | |
| **FACT-Hep** | 73.67(61.79-85.53) | 103.5 (-106.15-313.15) | 91.5 (34.32-148.67) | -^a^ |
| **FACT-G** | 53.67 (45.99-61.34) | 70.5 (-62.91-203.91) | 68 (68-68) | -^a^ |
| **TOI** | 51.5 (44.93-58.06) | 69.5 (-51.20-190.20) | 65 (1.47-128.53) | -^a^ |
| **HepCS** | 20 (12.20-27.79) | 33 (-43.23-109.23) | 23.5 (-33.67-80.67) | -^a^ |
| **PWB** | 8.67 (2.89-14.44) | 12.5 (6.14-18.85) | 15 (2.29-27.70) | -^a^ |
| **SWB** | 23.5 (10.5) | 24 (-26.82-74.82) | 26.5 (7.44-45.55) | -^a^ |
| **EWB** | 10 (2.34-17.65-) | 15 (15-15) | 6 (-19.41-31.41) | -^a^ |
| **FWB** | 12.17 (8.10-16.22) | 19 (-69.94-107.94-) | 20.5 (-11.26-52.26) | -^a^ |

-^a^**: n=1**
