# Supplementary material for: The first validation of the Functional Assessment of Cancer Therapy Hepatobiliary (FACT-Hep) for evaluating health-related quality of life (HRQOL) in patients with advanced-stage intrahepatic cholangiocarcinoma (biliary tract cancer)
Source: PLoS One. 2025 Apr 28;20(4):e0321618. doi: 10.1371/journal.pone.0321618 (PMC12036939; doi:10.1371/journal.pone.0321618)
Supplement: S4 Table — (DOCX) [file pone.0321618.s004.docx]

**Table S4 Comparison of FACT-Hep and its subscales in survivors and non-survivors on day 28 (1 month) and 56 (2 months) of treatment follow-up.**

| **Category** | **Survivors** | | **Non-survivors** | |
| --- | --- | --- | --- | --- |
|  | **1 month (28 days)** | **2 months (56 days)** | **1 month (28 days)** | **2 months (56 days)** |
| **Group 1** | | | | |
| **FACT-Hep** | 66 (49.93-82.06) | 73.5 (67.14-79.85) | 71.5 (-61.91-204.91) | - |
| **FACT-G** | 47.8 (33.71-61.88) | 50 (-0.82-100.82) | 63.5 (31.73-95.26) | - |
| **TOI** | 37 (26.13-47.86) | 49 (-1.82-99.82) | 39 (-37.23-115.23) | - |
| **HepCS** | 18.2 (12.34-24.05) | 23.5 (-33.67-80.67) | 13.5 (-18.26-45.26) | - |
| **PWB** | 5.6 (2.24-8.95) | 4 (-34.11-42.11) | 11 (-27.11-49.11) | - |
| **SWB** | 21 (12.81-29.18) | 20.5 (-36.67-77.67) | 28 (28-28) | - |
| **EWB** | 8 (2.80-13.19) | 4 (-8.70-16.70) | 4.5 (-52.67-61.67) | - |
| **FWB** | 13.2 (2.98-23.41) | 21.5 (-22.97-65.97) | 14.5 (8.14-20.85) | - |
| **Group 2** | | | | |
| **FACT-Hep** | 67.83 (53.15-82.51) | 75.33 (72.46-78.20) | 80 (-21.64-181.64) | - |
| **FACT-G** | 49.83 (41.77-57.89) | 58 (51.42-64.57) | 58 (19.88-96.11) | - |
| **TOI** | 40.67 (28.38-52.95) | 43.33 (39.53-47.12) | 44 (-121.180-209.180) | - |
| **HepCS** | 18 (10.23-25.76) | 17.33 (7.92-26.73) | 22 (-41.53-85.53) | - |
| **PWB** | 4.33 (-0.0021-8.66) | 6.67 (-6.08-19.41) | 8 (-17.41-33.41) | - |
| **SWB** | 22.33 (18.53-26.12) | 23.33 (12.99-33.67) | 27.5 (21.14-33.85) | - |
| **EWB** | 4.83 (0.40-9.25) | 8.67 (-2.53-19.86 ) | 8.5 (-48.67-65.67) | - |
| **FWB** | 18.33 (14.90-21.76) | 19.33 (-0.74-39.41) | 14 (-113.06-141.06) | - |
| **Group 3** | | | | |
| **FACT-Hep** | 73.5 (-98.03-245.03) | - | 79.67 (66.94-92.39) | 103.5 (-106.15-313.15) |
| **FACT-G** | 57.5 (-75.91-190.91) | - | 57.16 (48.32-66.00) | 70.5 (-62.91-203.91) |
| **TOI** | 51.5 (-56.50-159.50) | - | 56 (47.29-64.70) | 69.5 (-51.20-190.20) |
| **HepCS** | 16 (-22.11-54.11) | - | 22.5 (15.02-29.97) | 33 (-43.23-109.23) |
| **PWB** | 9 (-79.94-97.94) | - | 10.67(5.71-15.61) | 12.5 (6.14-18.85) |
| **SWB** | 26.5 (7.44-45.55) | - | 23.5 (10.5) | 24 (-26.82-74.82) |
| **EWB** | 4 (4-4) | - | 10.67 (3.52-17.80) | 15 (15-15) |
| **FWB** | 18 (-45.53-81.53) | - | 13 (8.213-17.78) | 19 (-69.94-107.94-) |
