# Supplementary material for: The first validation of the Functional Assessment of Cancer Therapy Hepatobiliary (FACT-Hep) for evaluating health-related quality of life (HRQOL) in patients with advanced-stage intrahepatic cholangiocarcinoma (biliary tract cancer)
Source: PLoS One. 2025 Apr 28;20(4):e0321618. doi: 10.1371/journal.pone.0321618 (PMC12036939; doi:10.1371/journal.pone.0321618)
Supplement: S1 File — (DOCX) [file pone.0321618.s005.docx]

**Supplementary Information**

**Methods**

**Inclusion and exclusion criteria**

Patient’s inclusion criteria were as follows: unresectable and/or metastatic iCCA confirmed by ultrasonography and contrast-enhanced computed tomography (CT) or Magnetic Resonance Cholangiopancreatography (MRCP), serum carcinoembryonic antigen (CEA) of > 5.2 ng/ml, and/or serum carbohydrate antigen 19-9 (CA19-9) of > 129 U/ml, measurable tumor (≥1 lesion) following the Response Evaluation Criteria in Solid Tumors (RECIST: version 1.1), Eastern Cooperative Oncology Group Performance State (ECOG) status of 0-2, normal cardiac function and electrocardiograms (ECGs), normal bone marrow and organ functions with polymorphonuclear cells (PMN) of> 1,500 cell/µl, platelets of>100,000 cells/µl, and normal hemoglobin levels, normal blood coagulation, no bleeding and no concurrent use of anticoagulants or antiplatelets, ability to communicate, refused chemotherapies, and obtainment of informed consent for study participation [33]. The exclusion criteria were pregnancy or lactating before chemotherapy, radiotherapy treatment, hypersensitivity or idiosyncratic reaction to any medicine or herbal product, current or previous history of diagnosis of other types of cancer within five years, abnormal gastrointestinal absorption, disease or condition associated with immune deficiency, or participation in any other study in the past three months [33].

Patients were randomized to receive treatment as follows: Group 1: daily dose of 1,000 mg the capsule formulation of Atractylodes Lancea (CMC-AL) for 90 days, with standard supportive care (n=15); Group 2: daily dose of 1,000 mg CMC-AL for 14 days, followed by 1,500 mg for 14 days, and 2,000 mg for 62 days, with standard supportive care (n=16); and Group 3: standard supportive care alone (n=16). Each CMC-AL capsule contained 2.45 mg and 4.06 mg of atractylodin and β-eudesmol, respectively. The drug was given in the morning, two hours before breakfast [33].

Patient’s withdrawal criteria included a life-threatening adverse event related to study drug, abnormality of blood coagulation or bleeding, Glasgow Coma Score of<15, increased in serum bilirubin and transaminase enzymes (AST and ALT) of> 1.5-fold baseline levels, serum creatinine > 1.5-fold baseline level or creatinine clearance < 50 ml/min, PMN of<1,500 cell/µl, platelets of< 100,000 cells/µl, and hemoglobin < 8.0 g/dl, or condition affecting study participation. Patient’s discontinuation criteria were those with significant protocol non-compliance or withdrawal of informed consent for study participation. The termination criterion for the study was a report of any suspected unexpected serious adverse reactions (SUSAR) and life-threatening reactions based on the physician’s opinion and death [33].
