# Supplementary material for: The first validation of the Functional Assessment of Cancer Therapy Hepatobiliary (FACT-Hep) for evaluating health-related quality of life (HRQOL) in patients with advanced-stage intrahepatic cholangiocarcinoma (biliary tract cancer)
Source: PLoS One. 2025 Apr 28;20(4):e0321618. doi: 10.1371/journal.pone.0321618 (PMC12036939; doi:10.1371/journal.pone.0321618)
Supplement: S2 File — (DOCX) [file pone.0321618.s006.docx]

| **MONTH ________ DAY _______** |
| --- |

**แบบสอบถามการประเมินคุณภาพชีวิตของผู้ป่วย**

**(Functional Assessment of Cancer Therapy – Hepatobiliary (FACT-Hep) version 4**

**คำชี้แจง**

ข้อความข้างล่างนี้เป็นข้อความที่ผู้ป่วยโรคเดียวกับท่านกล่าวว่ามีความสำคัญ ขอให้ท่านอ่านแล้วเลือกวงกลมเพียงหนึ่งหมายเลขในแต่ละหัวข้อ ตามความรู้สึกของท่าน **ในระยะ 7 วันที่ผ่านมาท่านรู้สึกว่าเป็นอย่างไรบ้าง**

|  | **ความผาสุกด้านร่างกาย** | **ไม่เลย** | **เล็กน้อย** | **ปานกลาง** | **ค่อนข้างมาก** | **มากที่สุด** |
| --- | --- | --- | --- | --- | --- | --- |
| GP1 | ข้าพเจ้ารู้สึกหมดเรี่ยวแรง .............................................................. | 0 | 1 | 2 | 3 | 4 |
| GP2 | ข้าพเจ้ามีอาการคลื่นไส้ ................................................................. | 0 | 1 | 2 | 3 | 4 |
| GP3 | ข้าพเจ้ามีอาการเจ็บป่วยจนไม่สามารถทำตามเป้าหมายของครอบครัวได้ .................................................................................. | 0 | 1 | 2 | 3 | 4 |
| GP4 | ข้าพเจ้ารู้สึกเจ็บปวด ...................................................................... | 0 | 1 | 2 | 3 | 4 |
| GP5 | ข้าพเจ้ารู้สึกมีอาการข้างเคียงจากยาที่รักษา.................................... | 0 | 1 | 2 | 3 | 4 |
| GP6 | ข้าพเจ้ารู้สึกเจ็บป่วย........................................................................ | 0 | 1 | 2 | 3 | 4 |
| GP7 | ข้าพเจ้ารู้สึกอยากนอนตลอดเวลา................................................... | 0 | 1 | 2 | 3 | 4 |

|  | **ความผาสุกด้านสังคม/ ครอบครัว** | **ไม่เลย** | **เล็กน้อย** | **ปานกลาง** | **ค่อนข้างมาก** | **มากที่สุด** |
| --- | --- | --- | --- | --- | --- | --- |
| GS1 | ข้าพเจ้ารู้สึกใกล้ชิดสนิทสนมกับเพื่อนๆ........................................ | 0 | 1 | 2 | 3 | 4 |
| GS2 | ข้าพเจ้าได้รับกำลังใจจากครอบครัว .............................................. | 0 | 1 | 2 | 3 | 4 |
| GS3 | ข้าพเจ้าได้รับความช่วยเหลือจากเพื่อนๆ ....................................... | 0 | 1 | 2 | 3 | 4 |
| GS4 | ครอบครัวของข้าพเจ้าเข้าใจและยอมรับได้ที่ข้าพเจ้าป่วย .............. | 0 | 1 | 2 | 3 | 4 |
| GS5 | ข้าพเจ้าพูดคุยกับสมาชิกในครอบครัวเกี่ยวกับอาการเจ็บป่วยได้อย่างเปิดใจ ................................................................................... | 0 | 1 | 2 | 3 | 4 |
| ขอให้ท่านอ่านแล้วเลือกวงกลมเพียงหนึ่งหมายเลขในแต่ละหัวข้อ ตามความรู้สึกของท่าน **ในระยะ 7 วันที่ผ่านมาท่านรู้สึกว่าเป็นอย่างไรบ้าง** | | | | | | |
|  | **ความผาสุกด้านสังคม/ ครอบครัว (ต่อ)** | **ไม่เลย** | **เล็กน้อย** | **ปานกลาง** | **ค่อนข้างมาก** | **มากที่สุด** |
| GS6 | ข้าพเจ้ารู้สึกสนิทสนมกับคู่ครอง (หรือคู่รัก)ของข้าพเจ้า ..... | 0 | 1 | 2 | 3 | 4 |
| Q1 | *หากท่านไม่ประสงค์จะตอบข้อต่อไปนี้ กรุณาทำเครื่องหมาย* × *ในช่องสี่เหลี่ยมนี้* ⬜  *และข้ามไปตอบหัวข้อถัดไป* | | | | | |
| GP7 | ข้าพเจ้ารู้สึกพอใจกับเรื่องทางเพศของข้าพเจ้า ............................... | 0 | 1 | 2 | 3 | 4 |

|  | **ความผาสุกด้านอารมณ์ จิตใจ** | **ไม่เลย** | **เล็กน้อย** | **ปานกลาง** | **ค่อนข้างมาก** | **มากที่สุด** |
| --- | --- | --- | --- | --- | --- | --- |
| GE1 | ข้าพเจ้ารู้สึกเศร้าใจ ....................................................................... | 0 | 1 | 2 | 3 | 4 |
| GE2 | ข้าพเจ้ารู้สึกพอใจกับวิธีที่ข้าพเจ้าปรับตัวกับการเจ็บป่วยของตนเอง ............................................................................................ | 0 | 1 | 2 | 3 | 4 |
| GE3 | ข้าพเจ้ารู้สึกท้อแท้ในการสู้กับโรคที่ข้าพเจ้าเป็น .......................... | 0 | 1 | 2 | 3 | 4 |
| GE4 | ข้าพเจ้ารู้สึกวิตกกังวล .................................................................... | 0 | 1 | 2 | 3 | 4 |
| GE5 | ข้าพเจ้ากังวลว่าอาจจะไม่มีชีวิตรอด .............................................. | 0 | 1 | 2 | 3 | 4 |
| GE6 | ข้าพเจ้ากังวลใจว่าอาการเจ็บป่วยจะแย่ลง ..................................... | 0 | 1 | 2 | 3 | 4 |

|  | **ความผาสุกด้านการปฏิบัติกิจกรรม** | **ไม่เลย** | **เล็กน้อย** | **ปานกลาง** | **ค่อนข้างมาก** | **มากที่สุด** |
| --- | --- | --- | --- | --- | --- | --- |
| GF1 | ข้าพเจ้าสามารถทำงานทั่วไปได้ (รวมถึงงานบ้าน) ....................... | 0 | 1 | 2 | 3 | 4 |
| GF2 | ข้าพเจ้าพึงพอใจในผลสำเร็จของงาน (รวมถึงงานบ้าน) ................ | 0 | 1 | 2 | 3 | 4 |
| GF3 | ข้าพเจ้ามีความสุขกับการใช้ชีวิต .................................................... | 0 | 1 | 2 | 3 | 4 |
| GF4 | ข้าพเจ้ายอมรับได้กับอาการเจ็บป่วยของข้าพเจ้า ........................... | 0 | 1 | 2 | 3 | 4 |
| GF5 | ข้าพเจ้าสามารถนอนหลับได้สนิท ................................................. | 0 | 1 | 2 | 3 | 4 |
| GF6 | ข้าพเจ้ายังคงมีความสุขกับสิ่งที่ข้าพเจ้าชอบทำ .............................. | 0 | 1 | 2 | 3 | 4 |
| GF7 | ข้าพเจ้าพอใจกับคุณภาพชีวิตของข้าพเจ้าตอนนี้ ............................ | 0 | 1 | 2 | 3 | 4 |

| ขอให้ท่านอ่านแล้วเลือกวงกลมเพียงหนึ่งหมายเลขในแต่ละหัวข้อ ตามความรู้สึกของท่าน **ในระยะ 7 วันที่ผ่านมาท่านรู้สึกว่าเป็นอย่างไรบ้าง** | | | | | | |
| --- | --- | --- | --- | --- | --- | --- |
|  | **ข้อคำถามเพิ่มเติม** | **ไม่เลย** | **เล็กน้อย** | **ปานกลาง** | **ค่อนข้างมาก** | **มากที่สุด** |
| C1 | ข้าพเจ้ามีอาการท้องบวมหรือเป็นตะคริวบริเวณท้อง..................... | 0 | 1 | 2 | 3 | 4 |
| C2 | ข้าพเจ้าน้ำหนักลดลง ..................................................................... | 0 | 1 | 2 | 3 | 4 |
| C3 | ระบบขับถ่ายของข้าพเจ้าทำงานได้เป็นปกติ ................................ | 0 | 1 | 2 | 3 | 4 |
| C4 | ระบบย่อยอาหารของข้าพเจ้าทำงานได้ดี ....................................... | 0 | 1 | 2 | 3 | 4 |
| C5 | ข้าพเจ้ามีอาการท้องเสีย ................................................................. | 0 | 1 | 2 | 3 | 4 |
| C6 | ข้าพเจ้ารับประทานอาหารได้ ........................................................ | 0 | 1 | 2 | 3 | 4 |
| Hep1 | ข้าพเจ้ารู้สึกไม่สบายใจเกี่ยวกับรูปร่างที่เปลี่ยนไป ........................ | 0 | 1 | 2 | 3 | 4 |
| CNS7 | ข้าพเจ้ารู้สึกปวดหลัง ..................................................................... | 0 | 1 | 2 | 3 | 4 |
| Cx6 | ข้าพเจ้ารู้สึกกังวลกับอาการท้องผูก ............................................... | 0 | 1 | 2 | 3 | 4 |

|  | **ข้อคำถามเพิ่มเติม** | **ไม่เลย** | **เล็กน้อย** | **ปานกลาง** | **ค่อนข้างมาก** | **มากที่สุด** |
| --- | --- | --- | --- | --- | --- | --- |
| HI7 | ข้าพเจ้ารู้สึกเหนื่อยล้า .................................................................... | 0 | 1 | 2 | 3 | 4 |
| An7 | ข้าพเจ้าสามารถทำกิจวัตรประจำวันได้ .......................................... | 0 | 1 | 2 | 3 | 4 |
| Hep2 | ข้าพเจ้ารู้สึกกังวลกับอาการซีดและผิวเหลืองของข้าพเจ้า .............. | 0 | 1 | 2 | 3 | 4 |
| Hep3 | ข้าพเจ้ารู้สึกมีไข้ (ตอนที่อุณหภูมิร่างกายสูง) ................................ | 0 | 1 | 2 | 3 | 4 |
| Hep4 | ข้าพเจ้ามีอาการคันตามผิวหนัง ...................................................... | 0 | 1 | 2 | 3 | 4 |
| Hep5 | ข้าพเจ้ามีการรับรสที่แปลกไป ....................................................... | 0 | 1 | 2 | 3 | 4 |
| Hep6 | ข้าพเจ้ามีอาการหนาวสั่น .............................................................. | 0 | 1 | 2 | 3 | 4 |
| HN2 | ข้าพเจ้ามีอาการปากแห้ง ............................................................... | 0 | 1 | 2 | 3 | 4 |
| Hep8 | ข้าพเจ้ามีอาการปวดท้องหรือไม่สบายท้อง .................................. | 0 | 1 | 2 | 3 | 4 |
