# Supplementary material for: The first validation of the Functional Assessment of Cancer Therapy Hepatobiliary (FACT-Hep) for evaluating health-related quality of life (HRQOL) in patients with advanced-stage intrahepatic cholangiocarcinoma (biliary tract cancer)
Source: PLoS One. 2025 Apr 28;20(4):e0321618. doi: 10.1371/journal.pone.0321618 (PMC12036939; doi:10.1371/journal.pone.0321618)
Supplement: S3 File — (DOCX) [file pone.0321618.s007.docx]

Below is a list of statements that other people with your illness have said are important**. Please circle or mark one number per line to indicate your response as it applies to the past 7 days.**

|  | **PHYSICAL WELL-BEING** | **Not at all** | | **A little bit** | **Some-what** | **Quitea bit** | **Very much** |
| --- | --- | --- | --- | --- | --- | --- | --- |
|  |  |  |  |  |  |  |  |
| GP1 | I have a lack of energy | 0 | | 1 | 2 | 3 | 4 |
| GP2 | I have nausea | 0 | | 1 | 2 | 3 | 4 |
| GP3 | Because of my physical condition, I have trouble meeting the needs of my family | 0 | | 1 | 2 | 3 | 4 |
| GP4 | I have pain | 0 | | 1 | 2 | 3 | 4 |
| GP5 | I am bothered by side effects of treatment | 0 | | 1 | 2 | 3 | 4 |
| GP6 | I feel ill | 0 | | 1 | 2 | 3 | 4 |
| GP7 | I am forced to spend time in bed | 0 | | 1 | 2 | 3 | 4 |
|  | | | | | | | |
|  | **SOCIAL/FAMILY WELL-BEING** | **Not at all** | | **A little bit** | **Some-what** | **Quitea bit** | **Very much** |
|  |  |  |  |  |  |  |  |
| GS1 | I feel close to my friends | 0 | | 1 | 2 | 3 | 4 |
| GS2 | I get emotional support from my family | 0 | | 1 | 2 | 3 | 4 |
| GS3 | I get support from my friends | 0 | | 1 | 2 | 3 | 4 |
| GS4 | My family has accepted my illness | 0 | | 1 | 2 | 3 | 4 |
| GS5 | I am satisfied with family communication about my illness | 0 | | 1 | 2 | 3 | 4 |
| GS6 | I feel close to my partner (or the person who is my main support) | 0 | | 1 | 2 | 3 | 4 |
| Q1 | *Regardless of your current level of sexual activity, please answer the following question. If you prefer not to answer it, please mark this box and go to the next section.* | |  |  |  |  |  |
| GS7 | I am satisfied with my sex life | | 0 | 1 | 2 | 3 | 4 |

**Please circle or mark one number per line to indicate your response as it applies to the past 7 days.**

|  | **EMOTIONAL WELL-BEING** | **Not at all** | | **A little bit** | **Some-what** | **Quitea bit** | **Very much** |
| --- | --- | --- | --- | --- | --- | --- | --- |
|  |  |  |  |  |  |  |  |
| GE1 | I feel sad | | 0 | 1 | 2 | 3 | 4 |
| GE2 | I am satisfied with how I am coping with my illness | | 0 | 1 | 2 | 3 | 4 |
| GE3 | I am losing hope in the fight against my illness | | 0 | 1 | 2 | 3 | 4 |
| GE4 | I feel nervous | | 0 | 1 | 2 | 3 | 4 |
| GE5 | I worry about dying | | 0 | 1 | 2 | 3 | 4 |
| GE6 | I worry that my condition will get worse | | 0 | 1 | 2 | 3 | 4 |

|  | **FUNCTIONAL WELL-BEING** | **Not at all** | | **A little bit** | **Some-what** | **Quitea bit** | **Very much** |
| --- | --- | --- | --- | --- | --- | --- | --- |
|  |  |  |  |  |  |  |  |
| GF1 | I am able to work (include work at home) | | 0 | 1 | 2 | 3 | 4 |
| GF2 | My work (include work at home) is fulfilling | | 0 | 1 | 2 | 3 | 4 |
| GF3 | I am able to enjoy life | | 0 | 1 | 2 | 3 | 4 |
| GF4 | I have accepted my illness | | 0 | 1 | 2 | 3 | 4 |
| GF5 | I am sleeping well | | 0 | 1 | 2 | 3 | 4 |
| GF6 | I am enjoying the things I usually do for fun | | 0 | 1 | 2 | 3 | 4 |
| GF7 | I am content with the quality of my life right now | | 0 | 1 | 2 | 3 | 4 |

**Please circle or mark one number per line to indicate your response as it applies to the past 7 days.**

|  | **ADDITIONAL CONCERNS** | **Not at all** | | **A little bit** | **Some-what** | **Quite**  **a bit** | **Very much** |
| --- | --- | --- | --- | --- | --- | --- | --- |
|  |  |  |  |  |  |  |  |
| C1 | I have swelling or cramps in my stomach area | | 0 | 1 | 2 | 3 | 4 |
| C2 | I am losing weight | | 0 | 1 | 2 | 3 | 4 |
| C3 | I have control of my bowels | | 0 | 1 | 2 | 3 | 4 |
| C4 | I can digest my food well | | 0 | 1 | 2 | 3 | 4 |
| C5 | I have diarrhea (diarrhoea) | | 0 | 1 | 2 | 3 | 4 |
| C6 | I have a good appetite | | 0 | 1 | 2 | 3 | 4 |
| Hep1 | I am unhappy about a change in my appearance | | 0 | 1 | 2 | 3 | 4 |
| CNS7 | I have pain in my back | | 0 | 1 | 2 | 3 | 4 |
| Cx6 | I am bothered by constipation | | 0 | 1 | 2 | 3 | 4 |
| H17 | I feel fatigued | | 0 | 1 | 2 | 3 | 4 |
| An7 | I am able to do my usual activities | | 0 | 1 | 2 | 3 | 4 |
| Hep2 | I am bothered by jaundice or yellow color to my skin | | 0 | 1 | 2 | 3 | 4 |
| Hep 3 | I have had fevers (episodes of high body temperature) | | 0 | 1 | 2 | 3 | 4 |
| Hep 4 | I have had itching | | 0 | 1 | 2 | 3 | 4 |
| Hep 5 | I have had a change in the way food tastes | | 0 | 1 | 2 | 3 | 4 |
| Hep 6 | I have had chills | | 0 | 1 | 2 | 3 | 4 |
| HN 2 | My mouth is dry | | 0 | 1 | 2 | 3 | 4 |
| Hep 8 | I have discomfort or pain in my stomach area | | 0 | 1 | 2 | 3 | 4 |
